# Dental Implant Surface Topography and Stability With Resonance Frequency Analysis: An Overview and Case Report

**DOI:** 10.7759/cureus.68725

**Published:** 2024-09-05

**Authors:** Nipun Dhalla, Pooja Palwankar, Lipika Gopal

**Affiliations:** 1 Periodontology, Manav Rachna Dental College, School of Dental Sciences, Manav Rachna International Institute of Research and Studies (MRIIRS), Faridabad, IND

**Keywords:** dental implants, implant stability quotient, osseointegration, resonance frequency analysis, surface topography and stability

## Abstract

Dental implants are fixtures that replace a natural tooth that has been missing. The outcome depends on the safety and longevity of the bone-implant relationship. The process of direct and strong anchoring of an implant due to the surrounding bone tissue growing around it is called osseointegration. The establishment of an osseointegrated contact depends on a variety of systemic and local variables and diagnostic methods. Resonance frequency analysis is one of the methods used to analyze implant stability. The surface topography, mainly surface texture and roughness, also helps in promoting a favorable interaction between the implant and biological tissues. This case report aimed to indicate the importance of implant surfaces showing primary and secondary stability and implant stability quotient (ISQ) values that can be analyzed by resonance frequency analysis (RFA) using the Osstell implant device, which can be a useful tool used to determine the risk of failure.

## Introduction

Oral rehabilitation of missing teeth with implant dentistry is a reliable beneficial approach with favorable long-term clinical outcomes. The primary stability of an implant is the fundamental factor that determines its success. Implant geometry, surface characteristics, quantity and quality of bone, surgical technique, and implant quality all can affect the implant's primary stability. Although the dentists cannot control the quantity or quality of bone loss at a given period of time, they can alter the geometry and surface of implants to improve primary stability and long-term implant treatment success [1].

Per-Ingvar Branemark established the concept of osseointegration as a reliable technique for securing implants for the support of dental prostheses. Ever since the osseointegration idea was introduced, the main focus of implant research has been on the characteristics of the interface between the implant and bone, as well as possible ways to increase the stability [2]. Osteointegration, another name for biologic stability, is the process by which new bone grows into the surface of the created osteotomy to support the dental implant, which increases with time. This occurs at two different stages: primary and secondary. The primary stability of an implant mainly comes from mechanical engagement with compact bone. Secondary stability, on the other hand, offers biological stability through bone regeneration and remodeling [3].

The physicochemical property and microstructure attributes of titanium implants can be modified by a variety of surface treatments, including sandblasting and acid etching, physical or chemical vaporizing anodization, oxidation, laser modifications, and cold spray, which can have an impact on the processes involved in bone formation. The process of sandblasting in conjunction with acid etching is the most often used treatment. This case report aimed to indicate the importance of implant surface showing primary and secondary stability and implant stability quotient (ISQ) values that can be analyzed by resonance frequency analysis (RFA) and also surface topography with an overview.

## Case presentation

A male patient, 42 years of age, reported to the Department of Periodontology, with a chief complaint of missing a lower anterior tooth for three months. He gave a history of extraction following a road traffic accident. Radiographical evaluation revealed adequate alveolar bone dimensions and bone quality for replacing the missing tooth with implants. A thorough medical history was recorded in order to rule out systemic issues and determine whether the patient has taken medicine for an extended period of time. A complete hematological investigation was performed prior to surgical intervention.

Surgical intervention 

**Figure 1:**
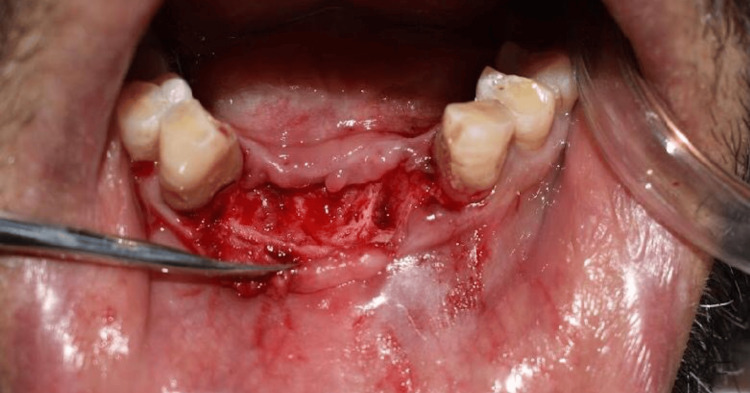
Flap reflection

After obtaining informed consent from the patient, local anesthesia, Xicaine 2% with adrenaline (one in 80,000) was administered, and a mid-crestal incision was made with a #15 blade to raise a full-thickness flap (Figure 1).

**Figure 2:**
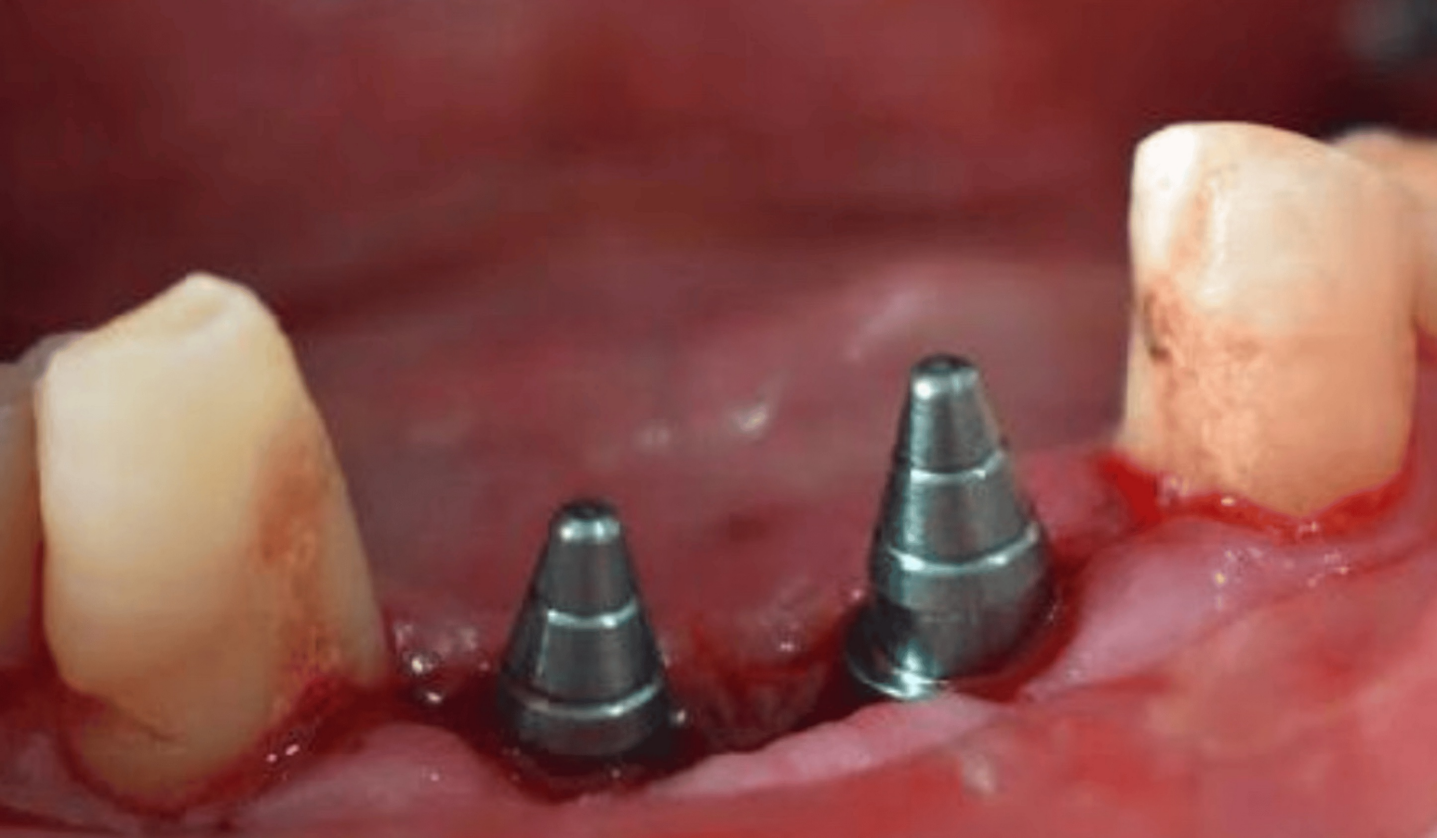
Placement of parallel pins

The osteotomy (32, 42 region) was done, and parallel pins were inserted to check the parallelism of the implants (Figure 2).

**Figure 3:**
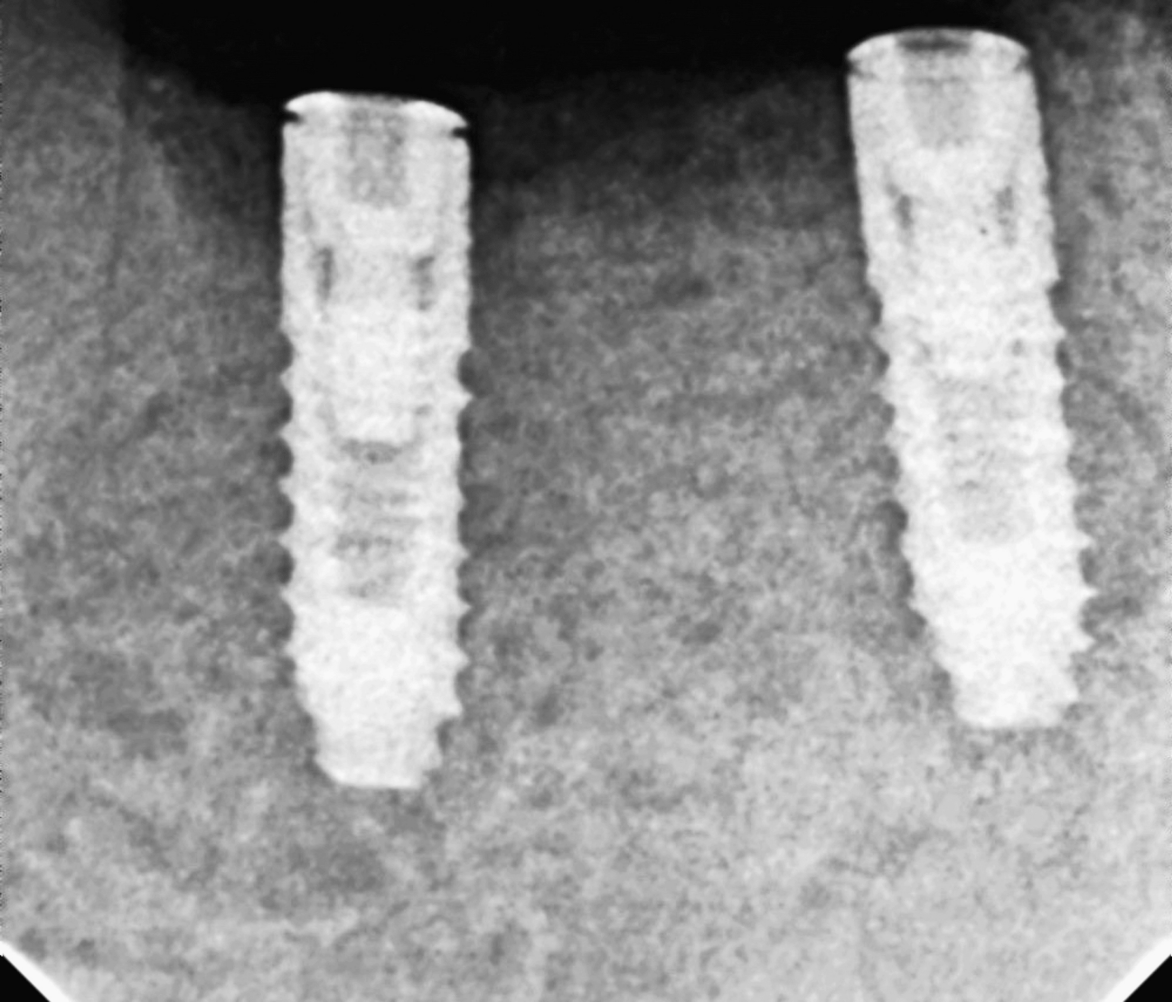
Radiograph after placement

As desired, osstem implants (sandblasting and acid-etching-treated rough surfaces) (Osstem Implant Company, Korea) of 3 x 11.5 mm were selected and placed, and radiographs were taken (Figure 3).

**Figure 4:**
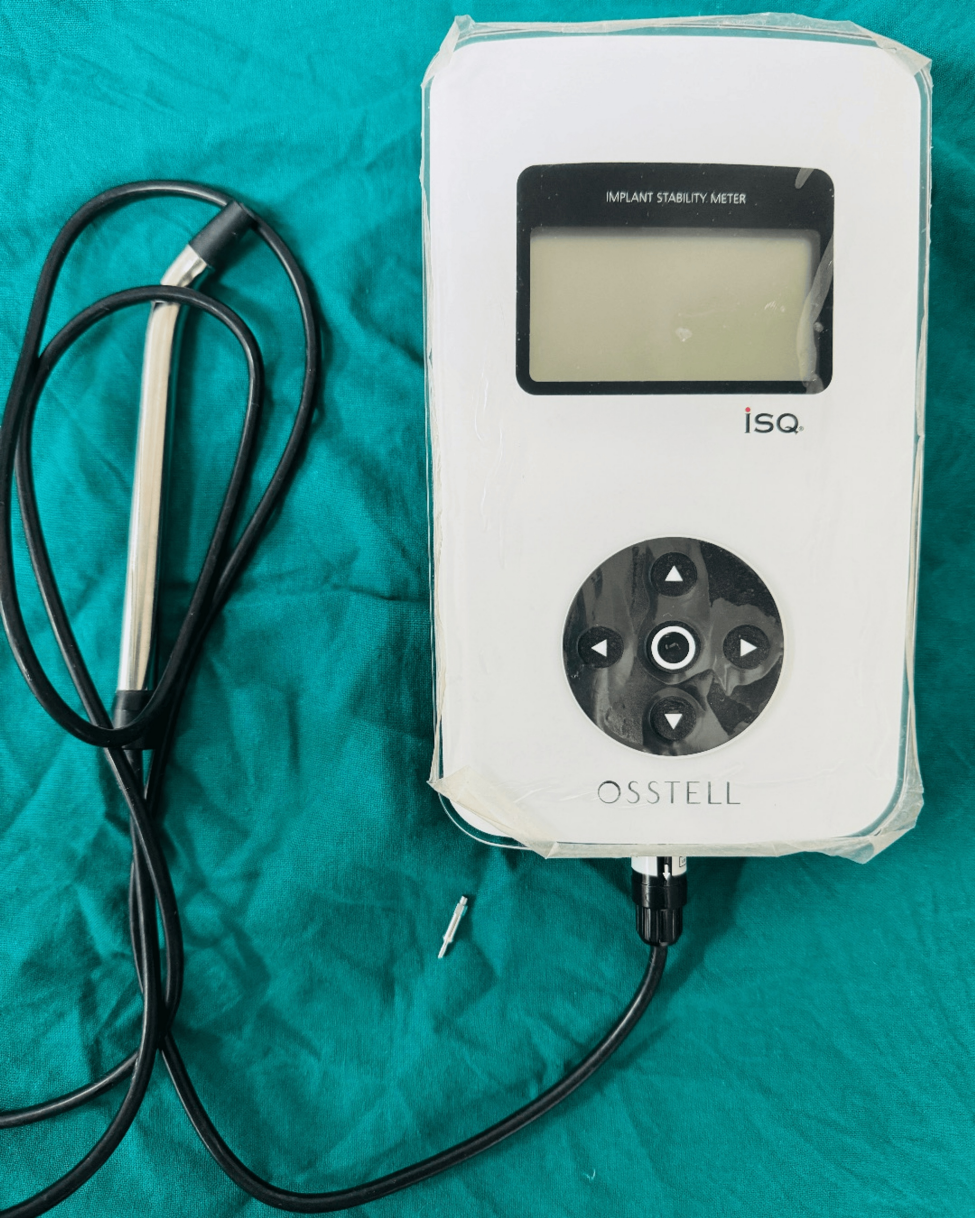
Resonance frequency analysis (RFA) device with a transducer

The primary stability of the implant was analysed using a resonance frequency analysis (RFA) (Osstell AB, Sweden) device (Figure 4).

**Figure 5:**
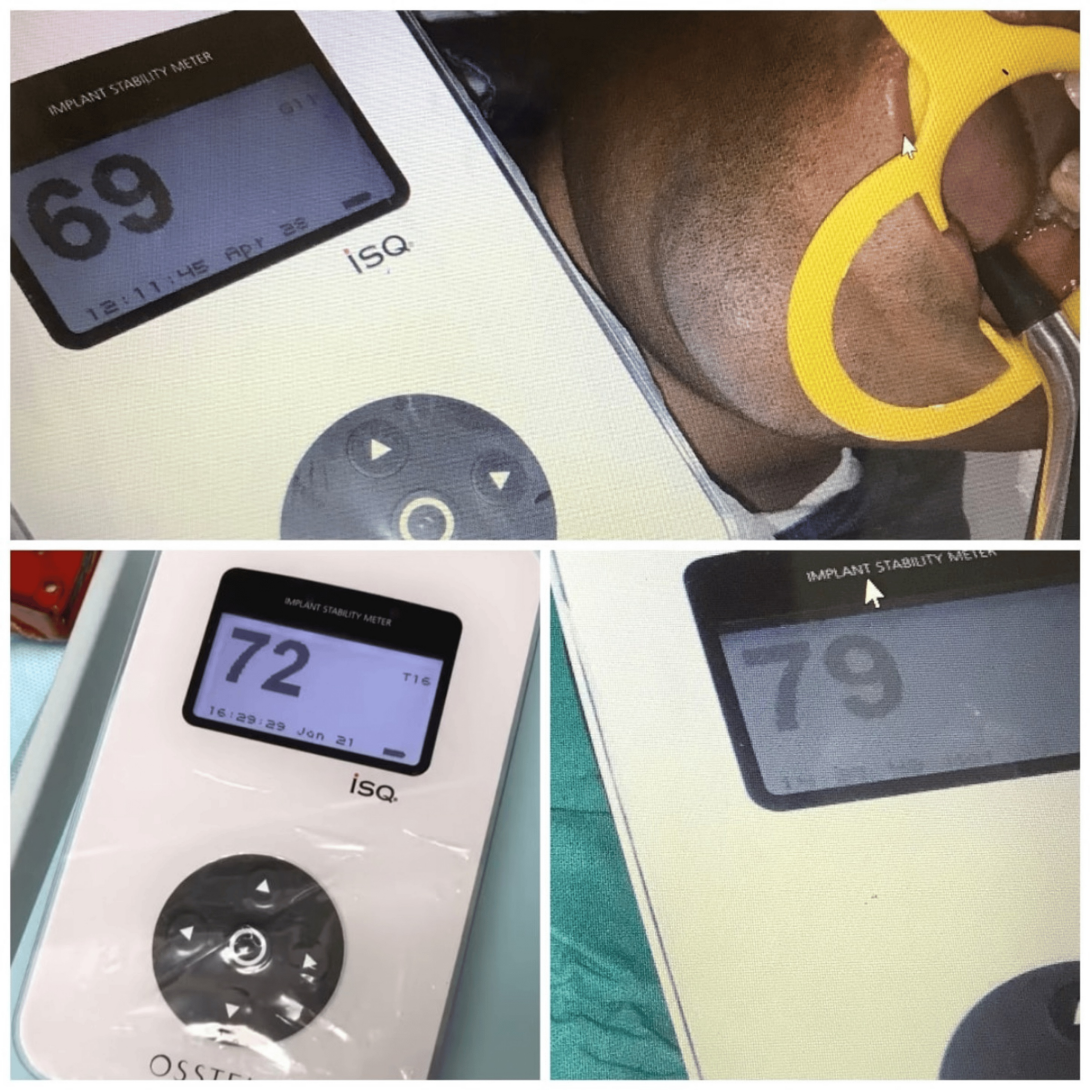
Resonance frequency analysis (RFA) analysis

The transducer (SmartpegTM, Osstell AB, Sweden) was fixed into the implant fixture to measure the ISQ values. After the final placement of implants, 3-0 silk sutures were placed, and the patient was recalled after one week for suture removal. Postoperative instructions were given to the patient. A follow-up after three months was performed to re-assess the stability of the implant using RFA (Figure 5).

**Figure 6:**
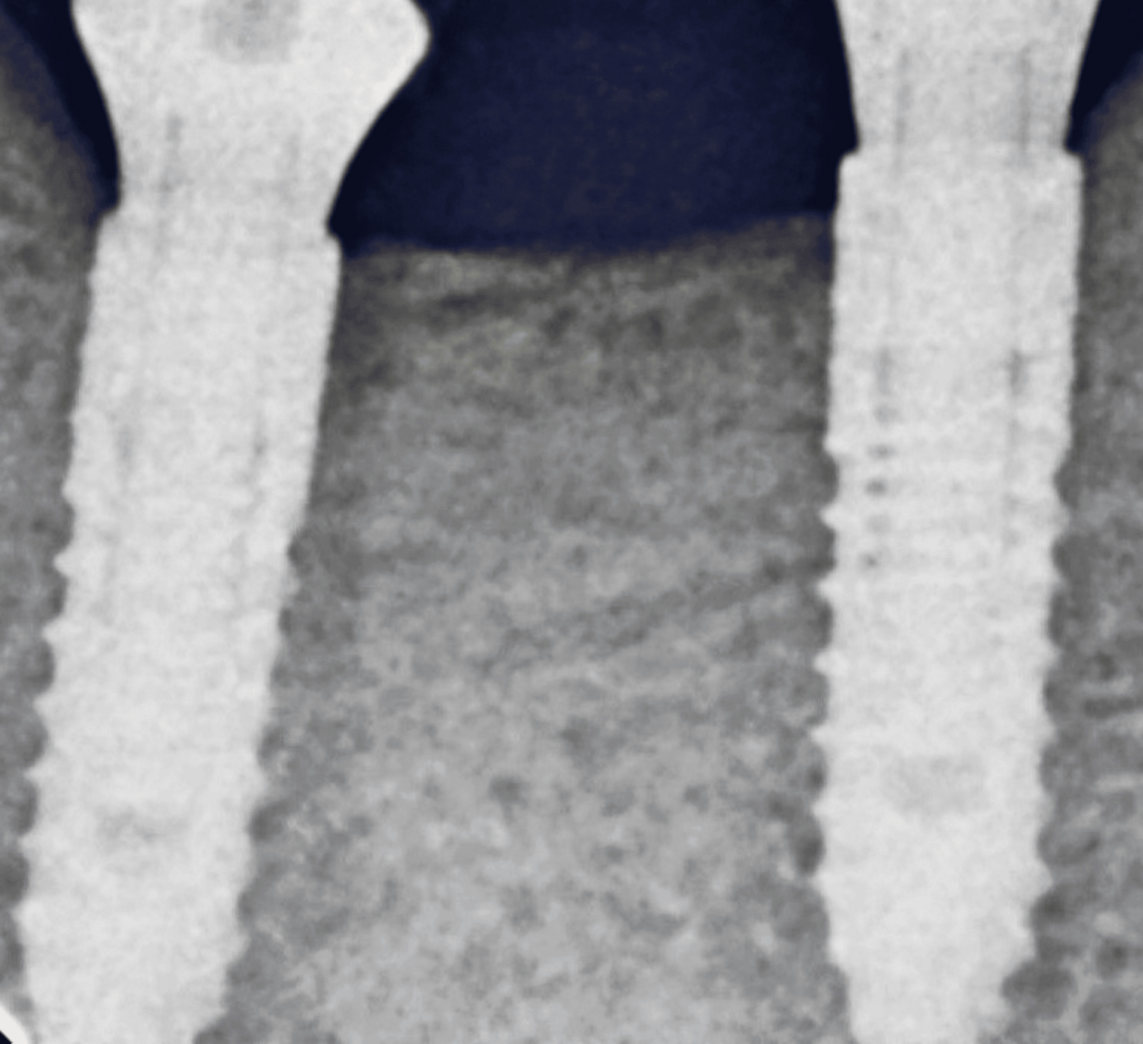
Postoperative radiograph after three months

**Figure 7:**
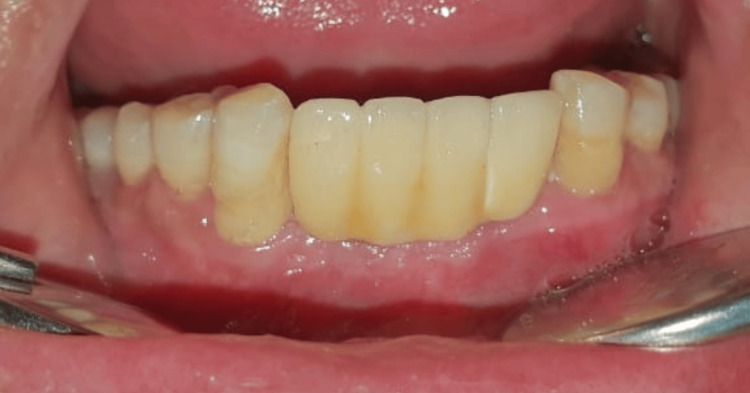
Placement of the prosthesis (three months)

The postoperative radiograph was taken (Figure 6) before the final placement of the prosthesis (Figure 7).

RFA

Immediately after implant placement, primary stability shows ISQ 67 and 69, and after three months, secondary stability shows ISQ 72 and 79. ISQ stands for implant stability quotient. There is an approximate correlation between the resonance frequency and the ISQ scale, which ranges from 1 to 100. The ISQ scale includes values of around 1,000 Hz as ISQ 1 and 10,000 Hz as ISQ 100. Relatively stable implants have ISQ values >60 [4]. Hence, it was found that surface roughness also plays an important role in the stability of the implant, along with other parameters.

## Discussion

In 1996, Meredith and colleagues successfully presented an overview of the RFA approach after several years of work [5]. This is a quantitative clinical approach that is objective and minimally invasive. It is used to gather valuable data for tracking implant success and osseointegration. Osstell was the one who designed the resonance frequency gadget that was first developed. It consisted of an L-shaped transmitter and a metallic transducer rod featuring a magnet on top that is attached to an implant and flashes a magnetic frequency. RFA measurements indicate the micro-mobility of dental implants [4].

The surface of dental implants has rapidly evolved in the last few years. Implants covered with thin films create direct contact between the metallic platform and the bone. It has been shown that the patterns of contact between the implant and bone govern the stability of the implant [6]. This surface interface's scope is extensive and encompasses multiple factors, which include the quality of bone, its quantity, stress caused by mechanical forces, surgical approach, and implant-related parameters including form, type, topography, and surface chemistry. This interface undergoes a complete transformation from its genesis (implantation into the prepared bone location) to its maturity (healing condition) [7]. Nanoscale topography features on the implant surface that affect protein adsorption and osteoblastic adhesion promote stability and a favorable environment for healing. The implant surface's acid etching produces a nanotopography that promotes bone formation, therefore raising the surface roughness. This was concluded by using two different implants with identical micro designs but different surface topographies. RFA measurements were made at two, four, six, and 12 weeks, which shows higher stability for the implants with a rough surface when compared with a smooth surface [8].

Implant dentistry frequently uses sandblasting and acid-etching (SLA) to increase the material's rugosity. Large-grit granules (alumina) in the 250-500 µm size range develop an SLA surface. After that, the surface gets chemically modified using an acid, such as nitric, hydrochloric, or sulphuric acid. This delivers roughness that contains big dips, sharp edges, and small micropits to enhance the contact surface for osseointegration [9]. A study was done showing the comparison between sandblasted acid-etched and oxidized titanium implant surfaces on six rabbit models. The roughness was analyzed by an interferometric microscope for residual stress load on the surfaces. Results revealed that SLA surfaces show slightly higher residual stress when compared with oxidized titanium implants, which show more hard tissue and good anchorage [9].

Implant stability is a clinical assessment of an implant's immobility and an indirect indicator of osseointegration. Many diagnostic investigations, including modal evaluation, torque resistance testing, implant stability standardized imaging, and resonance frequency analysis (RFA), are currently indicated.

According to this case report, the sandblasting and acid-etching treated rough surface of the implant shows better osteointegration over a period of time, as immediately after placement, the primary stability of ISQ 67 and 69 was noticed, which after three months showed higher stability of ISQ 72 and 79, respectively, by RFA using the Osstell Implant device.

Research has shown that ISQ measurements can provide the clinician with valuable information about the present state of the bone-implant interface. Kim MJ et al. did a study to determine the initial and secondary stability of the implants using ISQ values. The average initial stability was 67 ISQ and secondary stability was 80 ISQ on the implant with acid-etched surface sandblasted with alumina, showing 94.8% of survival rates. [10]. Another study concluded that the RFA score for implant stability of implant placement was found to be higher (at the end of three months) in the anterior segment of the mandible [11]. A study was conducted to measure the stability and crestal bone level changes in a freshly extracted socket, RFA was done at zero, three, six, and 12 months and radiographs at zero, six, and 12 months. The mean RFA obtained was 48.0 ISQ, 52.08 ISQ, 60.53 ISQ, and 66.32 ISQ, and the mean bone loss at 12 months was 0.67 mm. Hence, they proved that stability on implants improves with duration and type of implant used and RFA is a reliable method to analyze the stability [12]. 

## Conclusions

Dental implants are the best alternative for replacing missing teeth. Implants can be of various sizes, surfaces, and lengths, each with unique surface characteristics. Dental implants are highly dependent on surface topography, which is categorized into three levels: macrotopography, microtopography, and nanotopography. Surface roughness affects both the quality and osseointegration rate of dental implants made of titanium. It has been demonstrated that highly roughened implants promote mechanical anchoring and primary fixation to bone.

At any point after implant implantation, the RFA approach delivers clinically significant data regarding the condition of the implant-bone interface. An implant's micro-mobility when loaded is measured in its ISQ value, which is based on the integrity of the bone-implant interface and the biomechanical characteristics of the bone and surrounding tissue. Therefore, the selection and placement of dental implants require a thorough understanding of surface topography.
